# Supplementary material for: Time‑resolved multi-omic analysis of paclitaxel exposure in human iPSC‑derived sensory neurons unveils mechanisms of chemotherapy‑induced peripheral neuropathy
Source: Cell Death Dis. 2026 Feb 10;17(1):211. doi: 10.1038/s41419-026-08445-2 (PMC12921266; doi:10.1038/s41419-026-08445-2)
Supplement: Supplementary file 15 — Dataset 1. [file 41419_2026_8445_MOESM15_ESM.zip › CDD_Open_Data/04_RNA_Seq/Timeline_panel_plots_etc_2023-01-23_1609.zip!/Panel_Plots_etc/Evidence_Plots/24h/evidence_plot_standard contrasts_msigdb_hallmark_24h_PTX_vs_DMSO_ID4_M5890_HALLMARK_TNFA_SIGNALING_VIA_NFKB.pdf]

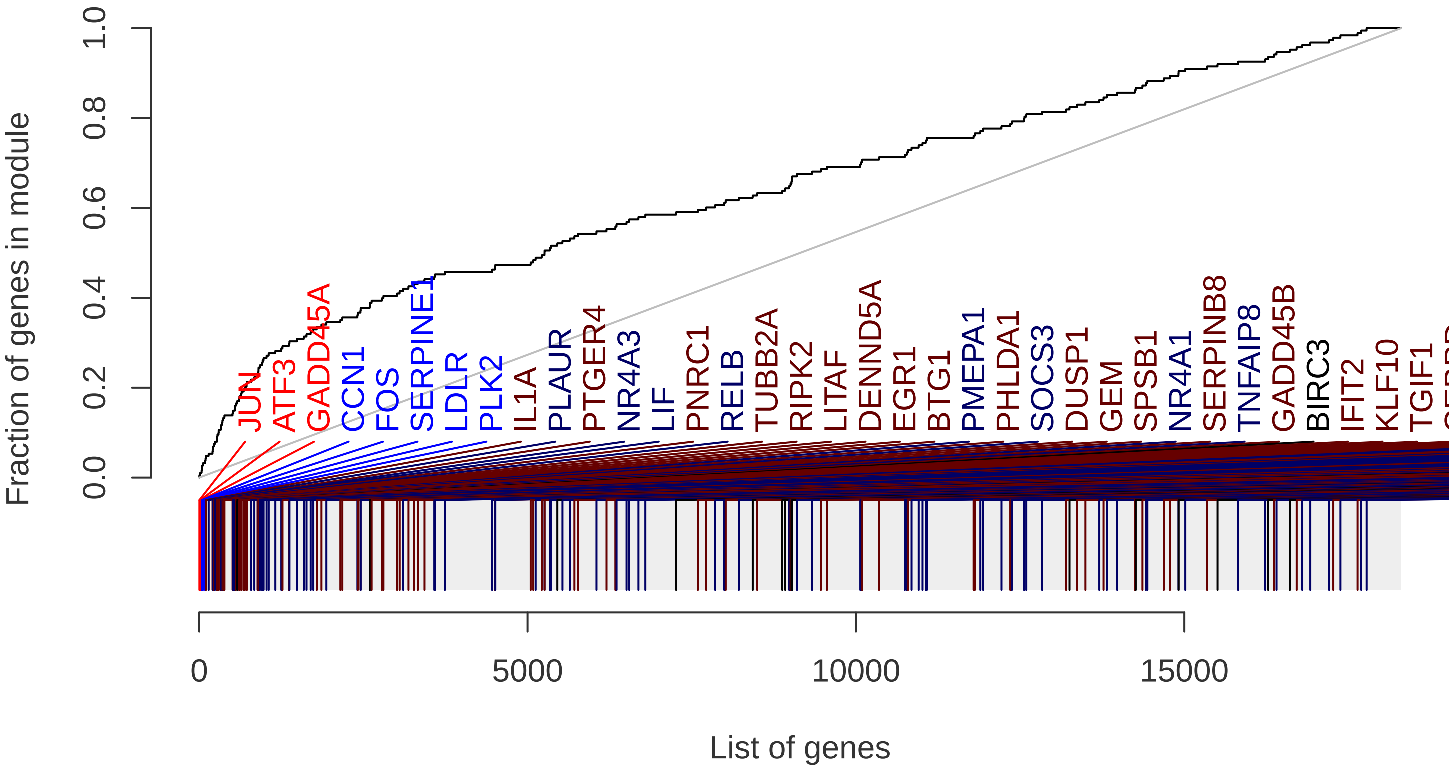

Fraction of genes in module
0.0
0.2
0.4
0.6
0.8
1.0
List of genes
0
5000
10000
15000
JUN
ATF3
GADD45A
CCN1
FOS
SERPINE1
LDLR
PLK2
IL1A
PLAUR
PTGER4
NR4A3
LIF
PNRC1
RELB
TUBB2A
RIPK2
LITAF
DENND5A
EGR1
BTG1
PMEPA1
PHLDA1
SOCS3
DUSP1
GEM
SPSB1
NR4A1
SERPINB8
TNFAIP8
GADD45B
BIRC3
IFIT2
KLF10
TGIF1
